# Do proinflammatory cytokines play a role in clozapine-associated glycometabolism disorders?

**DOI:** 10.1007/s00213-021-05824-9

**Published:** 2021-03-27

**Authors:** Tongtong Zhao, Kai Zhang, Yelei Zhang, Yating Yang, Xiaoshuai Ning, Yu Hu, Xiaoyue Li, Yulong Zhang, Lei Xia, Zhenhua Ren, Huanzhong Liu

**Affiliations:** 1 Department of Psychiatry, Chaohu Hospital of Anhui Medical University, 64 North Chaohu Road, Hefei, Anhui Province China; 2 Anhui Psychiatric Center, Anhui Medical University, Hefei, Anhui Province China; 3 Department of Anatomy, Anhui Medical University, Road Hefei, Meishan, 81 Anhui Province China

**Keywords:** Clozapine, Proinflammatory cytokine, Glycometabolism disorders, Immunomodulatory

## Abstract

**Rationale and objective:**

Clozapine (CLZ) is the most effective drug for treatment-resistant schizophrenia but is associated with many side effects, including glycometabolism disorders. Immunological mechanisms may be involved in the development of clozapine side effects. Research relating the immunomodulatory effects of clozapine and its early markers to clinically relevant adverse events is needed to reduce the harmful side effects of clozapine. This study aimed to investigate the role of proinflammatory cytokines in clozapine-associated glycometabolism disorders.

**Methods:**

We measured the effect of a range of doses of clozapine on glycometabolism-related parameters and proinflammatory cytokines levels in mice peripheral blood. We also examined the differences between these indicators in the peripheral blood of clozapine-treated schizophrenia patients and healthy controls. Furthermore, we detected proinflammatory cytokines expression in mice pancreatic tissue.

**Results:**

Following clozapine administration, glucagon significantly decreased in mouse serum, and proinflammatory cytokine IL-β levels markedly increased. Clozapine reliably increased proinflammatory cytokines (IL-1β, IL-6, and TNF-α) expression in murine pancreatic tissue. Compared with healthy controls, clozapine-treated patients’ BMI, blood glucose, and proinflammatory cytokines (IL-1β, IL-6, and TNF-α) increased significantly. In clozapine-treated patients, a higher clozapine daily dosage was associated with higher levels of the proinflammatory cytokines IL-1β and IL-6, and a significant positive correlation was observed between blood glucose levels and the proinflammatory cytokines IL-6 and TNF-α.

**Conclusion:**

Findings from animal experiments and clinical trials have shown clear evidence that clozapine has a regulatory effect on immune-related proinflammatory cytokines and influences glycometabolism indicators.

## Introduction

Schizophrenia is a serious mental disease that causes obstacles in thinking, emotion, and behavior, affecting approximately 1% of the world’s population (Insel 2010). More than 30% of individuals diagnosed with schizophrenia have treatment-resistant schizophrenia (Lally and MacCabe 2015). Second-generation antipsychotics (SGAs) are currently the first-line treatment for schizophrenia. Clozapine is the only antipsychotic drug approved for the treatment of treatment-resistant schizophrenia and is one of the most effective antipsychotics (Tiihonen et al. 2017). Numerous clinical reports have linked SGAs, especially clozapine, to severe metabolic side effects, including weight gain, obesity, and diabetes mellitus (Melkersson and Dahl 2003). However, the mechanisms by which clozapine induces glycometabolism disorders remain unclear and debatable, which, in turn, largely restricts clozapine as a maintenance treatment against refractory schizophrenia.

Recent studies have proposed type 2 diabetes as an inflammatory disease, and glucose metabolism disorder itself is also a state of inflammation (Donath and Shoelson 2011). Studies have shown that proinflammatory cytokines are an important part of the early inflammatory response to diabetes (Donath et al. 2019), and clinical research has found that the circulating levels of acute-phase proteins, cytokines, and chemokines in type 2 diabetes patients are elevated (Herder et al. 2005; Van Dyke et al. 2017; Volpe et al. 2014). Furthermore, some studies have suggested that the subclinical inflammatory reaction precedes the onset of type 2 diabetes. Increased levels of interleukin-1β (IL-1β), IL-6, and tumornecrosis factor-α (TNF-α) in peripheral blood can predict the occurrence of type 2 diabetes in patients (Mirza et al. 2012; Pham et al. 2011; Pradhan et al. 2001; Spranger et al. 2003), indicating that patients may already be in the early stage of glucose metabolism disorders. The vasculature, as well as other tissues and organs, undergoes immune regulation and inflammation. In a diabetes animal model, the proinflammatory cytokines IL-6, TNF-α, and IL-1β increased significantly in rat pancreatic tissue (Hsiao et al. 2019). In summary, these studies indicate that proinflammatory cytokines are involved in the development of diabetes metabolic disorders. Clozapine has a well-known and significant immunomodulatory effect (Baumeister et al. 2016; Røge et al. 2012). Studies have shown that clozapine can induce a generalized inflammatory response in the first 2 weeks of treatment, which is characterized by increased peripheral blood proinflammatory cytokines and C-reactive protein. During this period, patients are suspected of being at the highest risk of drug-related side effects (Verdoux et al. 2019). Notably, immune-mediated mechanisms have been proposed for the side effects of clozapine that cause metabolic dysregulation and weight gain (Chen et al. 2017). One study reported that proinflammatory cytokines levels are elevated in female schizophrenia patients treated with clozapine, wherein IL-1β could be related to glycometabolism disorders and weight gain (O’Connell et al. 2014). The latest report showed that patients with schizophrenia who are treated with clozapine or olanzapine alone and have metabolic problems have significantly higher plasma IL-6 and TNF-α levels than healthy controls (Fang et al. 2020).

Immunometabolism via metabolic stress can cause pathologic activation of the immune system and has revealed new insights into the pathogenesis and progression of diabetes mellitus (Hameed et al. 2015). Clozapine is both the most prominent antipsychotic with metabolic problems and an agent with significant immunomodulatory effects. Therefore, this study aimed to investigate the role of proinflammatory cytokines in clozapine-related immune and glycometabolism disorders. We used animal model experiments and clinical trials to evaluate the effects of clozapine on the expression of proinflammatory cytokines and glycometabolism parameters.

## Materials and methods

### Animal model

Eight-week-old male C57BL/6 mice were obtained from the experimental animal center of Anhui Medical University. All mice were maintained in a 12-h/12-h light/dark cycle at a temperature of 22±2 °C and relative humidity of 60 ± 5%, with free access to standard chow and water. After a week of acclimation, forty-eight mice were randomly divided into four groups (*n* = 12 per group): clozapine 5 mg/kg, clozapine 10 mg/kg, clozapine 20 mg/kg, and saline. The clozapine used in the study was purchased from Shanghai Shangyao Xinyi Pharmaceutica Co., Ltd. (National Drug Approval Number: H31021152). Mice were treated with clozapine 5, 10, and 20 mg/kg or an equal volume of saline by gavage at 9:00 am every day for 14 consecutive days. Body weight was measured before and after the experiment. After the mice fasted for 12 h after the final intragastric administration, blood glucose concentrations were measured in whole blood collected from the tail vein using One Touch Ultra glucometers (Lifescan Technologies, Milpitas, CA, USA). Then, the mice were anesthetized by intraperitoneal injection of chloral hydrate, and blood samples were immediately collected by cardiac puncture. The pancreases of the mice were dissected, immediately frozen in dry ice, and then stored at −80 °C. The serum was separated from the blood samples and stored at −80 °C for measurement. The experiments were approved by the local ethics committees of Anhui Medical University (LLSC20190743), and all efforts were made to minimize the number of animals used and to reduce suffering.

### Measurement of peripheral blood parameters in animal experiments

Mice serum was separated and stored at −80 °C for ELISA. The amylase levels in the serum were measured using the amylase assay colorimetric kit (ab102523) from Abcam (Cambridge, UK), and the level of amylase was recorded in mU/ml (nmol/min/ml). The serum glucagon levels were assessed using the glucagon ELISA kit (DGCG0) from R&D Systems (Minneapolis, MN, USA). Glucagon values are presented in pg/ml. Serum insulin levels were calculated using a commercial insulin ELISA kit (ab100578) from Abcam (Cambridge, UK). Insulin values are presented in μlU/ml. ELISA kits (ab197742, ab222503, and ab46105) for detection of the proinflammatory cytokines IL-1β, IL-6, and TNF-α were obtained from Abcam (Cambridge, UK) and used according to the manufacturer’s instructions, and the levels of IL-1β, IL-6, and TNF-α were recorded in pg/ml.

### Measurement of proinflammatory cytokines protein expression in pancreatic tissue in animal experiments

Proinflammatory cytokines protein expression in the mice pancreas was evaluated by western blot analysis. Six pancreatic tissue samples were taken from each of the four groups. Each sample of pancreatic tissue was homogenized in RIPA buffer (Beyotime Biotechnology, Shanghai, CN), and protein concentrations were determined with the BCA Protein Assay Kit (Beyotime Biotechnology, Shanghai, CN). Proteins were separated by SDS-PAGE and electrophoretically transferred onto PVDF membranes (Millipore Corporation, Billerica, MA, USA). Then, membranes were blocked and incubated with primary antibodies for 4 h at room temperature with gentle shaking. The membrane was then washed 3 times with TBST, incubated with secondary antibody for 2 h, and washed 3 more times. Immune complexes were detected with an enhanced chemiluminescent substrate (Thermo Fisher Scientific, Rockford, IL, USA). Immunoblotting densities were quantified with ImageJ software. The materials for western blot analysis were obtained from the following suppliers. Mouse α-tubulin antibody (T6199) was purchased from Sigma-Aldrich (St. Louis, MO, USA). Rabbit IL-1β and TNF-α antibodies (ab9722 and ab6671) were purchased from Abcam (Cambridge, UK). Rabbit IL-6 antibody (12912S) was purchased from Cell Signaling Technology (Danvers, MA, USA). HRP-conjugated goat anti-rabbit and goat anti-mouse secondary antibodies were purchased from Zs-BIO (Zs-BIO, Beijing, CN).

### Measurement of proinflammatory cytokines mRNA expression in pancreatic tissue in animal experiments

The mRNA expression of proinflammatory cytokines in the mice pancreas was determined by quantitative real-time polymerase chain reaction (qRT-PCR) using species-specific primers. Six pancreatic tissue samples were taken from each of the four groups, and each sample of pancreatic tissue RNA was extracted with TRIzol (Life Technologies, Grand Island, NY, USA) and then dissolved in DEPC-H2O (Generay Biotech, Shanghai, CN). cDNA was synthesized from total RNA using a RevertAidTM First Strand cDNA Synthesis Kit (Thermo Scientific, Waltham, MA, USA) under the conditions recommended by the manufacturer. Real-time PCR analysis was performed using Novostart SYBR qPCR SuperMix Plus (Novoprotein, Shanghai, CN). The real-time PCR conditions were as follows: initial amplification step for 1 min at 95 °C, followed by 40 cycles of denaturation for 20 s at 95 °C and annealing for 1 min at 60 °C. Data were collected using the Applied Biosystems StepOne Plus Real-Time PCR system (Thermo Scientific, Waltham, MA, USA) to determine target gene expression (Wang et al. 2019). The expression levels of the target gene were quantified by comparison with a standard curve and normalized using the expression levels of β-actin as a housekeeping gene. Primer sequences were synthesized by Sangon Biotech Co., Ltd. (Table 1).

**Table 1:** Nucleotide sequence of IL-1β, IL-6, and TNF-α

Gene	Nucleotide sequence
IL-1β	F: 5′-GAAGAAGAGCCCATCCTCTG-3′ R: 5′-TCATCTCGGAGCCTGTAGTG-3′
IL-6	F: 5′-AGTCCGGAGAGGAGACTTCA-3′ R; 5′-ATTTCCACGATTTCCCAGAG-3′
TNF-α	F: 5′-GACAGTGACCTGGACTGTGG-3′ R: 5′-TGAGACAGAGGCAACCTGAC-3′
β-actin	F: 5′-AGTGTGACGTTGACATCCGT-3′ R: 5′-TGCTAGGAGCCAGAGCAGTA-3′

### Subjects in the clinical trial

We investigated twenty male patients with schizophrenia who were treated with clozapine and twenty age-matched male healthy controls. Patients with schizophrenia were recruited if they met the following criteria: (1) aged 20–60 years; (2) met the criteria of the 10th revision of the International Classification of Diseases (ICD-10); (3) stabilized on clozapine monotherapy; and (4) did not meet the ICD-10 criteria for serious mental illnesses other than schizophrenia, such as major depressive disorder and obsessive-compulsive disorder, as assessed by two senior psychiatrists. Based on the principle of voluntary participation, healthy controls were recruited from the community. The inclusion criteria for the community participants were as follows: (1) aged 20–60 years and (2) without severe neurological disease, intellectual disability, or serious mental disorders, such as schizophrenia. The exclusion criteria for both groups were as follows: (1) recent (in the last 3 months) critical illness known to be associated with immunological disease (e.g., hyperpyrexia, serious infections, acute organic abnormalities, and severe central nervous system disease); (2) recent use of medications that affect immune parameters, such as nonsteroidal anti-inflammatory drugs and immunomodulators; (3) substance or alcohol abuse. The study protocol was approved by the Human Research and Ethics Committee of Chaohu Hospital affiliated with Anhui Medical University (201805-kyxm-03), and all the study procedures were in line with the Declaration of Helsinki. Written informed consent was obtained from all individuals after a detailed description of the study.

### Measurement of peripheral blood parameters in the clinical trial

For all subjects, 10 mL of venous blood was collected between 07:00 AM and 08:00 AM after an overnight fast. Blood samples were collected into EDTA vacutainer tubes, and plasma was separated by centrifugation and stored at −80 °C until analysis, during which the technicians were blinded to subject status. The height and the weight of each subject were scaled using a normative standard, and BMI was calculated with the following formula: BMI = weight (kg)/height^2(m). Plasma levels of the proinflammatory cytokines IL-1β, IL-6, and TNF-α (Cat. No: 558279, 558276 and 558273) were assessed in duplicate by the cytometric bead array method (BD Biosciences, San Diego, CA, USA) using reagents from a single lot. The acquisition was performed with a BD LSRFortessa™ flow cytometer (BD Biosciences, San Jose, USA) (Kalmady et al. 2018). The plasma amylase level was detected using an automatic biochemistry analyzer with the EPS substrate method (Leadman Biotech, Beijing, China). Fasting blood glucose was measured by the oxidase method (Medical System Biotech, Zhejiang, China). Insulin was measured by electrochemiluminescence immunoassay (Roche Diagnostics, Shanghai, China). Glucagon was measured by radioimmunoassay (Beijing North Institute of Biotechnology, Beijing, China).

### Statistical analysis

SPSS version 23.0 (SPSS Inc. IBM, Chicago, IL, USA) was used for data analysis. The normality of the distribution was tested by the Kolmogorov–Smirnov one-sample test, and all variables were normally distributed. Numerical variables were represented as the means ± standard deviation except where otherwise stated. In animal experiments, a paired-samples *t*-test was applied to analyze the difference in each group before and after the experiment. One-way analysis of variance (ANOVA) was used to compare the sample means across the four mouse groups. The least significant difference (LSD) method was used when the variance was homogeneous, and the Dunnett T3 method was used when the variance was heterogeneous. In human subjects, differences in numerical variables between two groups (the clozapine group and control group) were analyzed using independent samples *t*-tests, and Pearson correlation analysis was used to analyze the correlation of the clozapine dosage/duration and glucose metabolism-related parameters with proinflammatory cytokine levels in the clozapine-treated patient group. GraphPad Prism version 6 software package (GraphPad Software, San Diego, CA, USA) was used to generate the graphs. The statistically significant values are marked with an asterisk.

## Results

### Animal experiments

#### The effect of clozapine on body weight

**Fig. 1:**
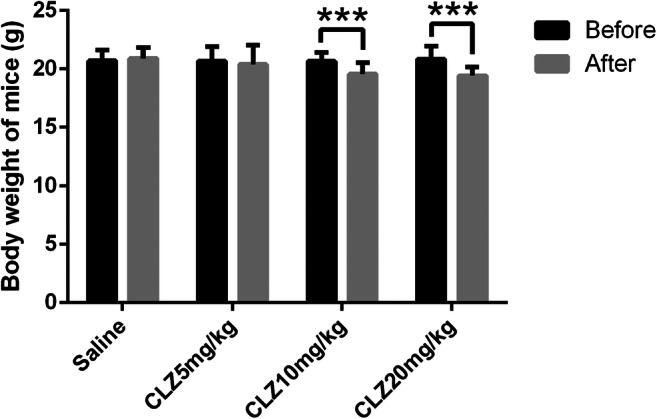
Body weight (g) before and after the experiment in groups of mice receiving daily intragastric administration of the indicated doses of clozapine for 14 days. A triple asterisk denotes significant difference (*P* < 0.001) compared to body weight before clozapine treatment. Variables are expressed as the means ± standard deviation (*n* = 12)

The body weights of the mice were measured before and after the experiment. After receiving daily intragastric administration of the indicated doses of clozapine for 14 days, mice treated with clozapine showed a significant decrease in body weight at the higher doses (10 and 20 mg/kg) compared with the values observed before clozapine treatment (all *P* < 0.001). The bodyweight of the mice in the 5 mg/kg clozapine group also decreased, but these decreases were not significantly different (Fig. 1).

#### The effect of clozapine on amylase, blood glucose, insulin, and glucagon levels

**Fig. 2:**
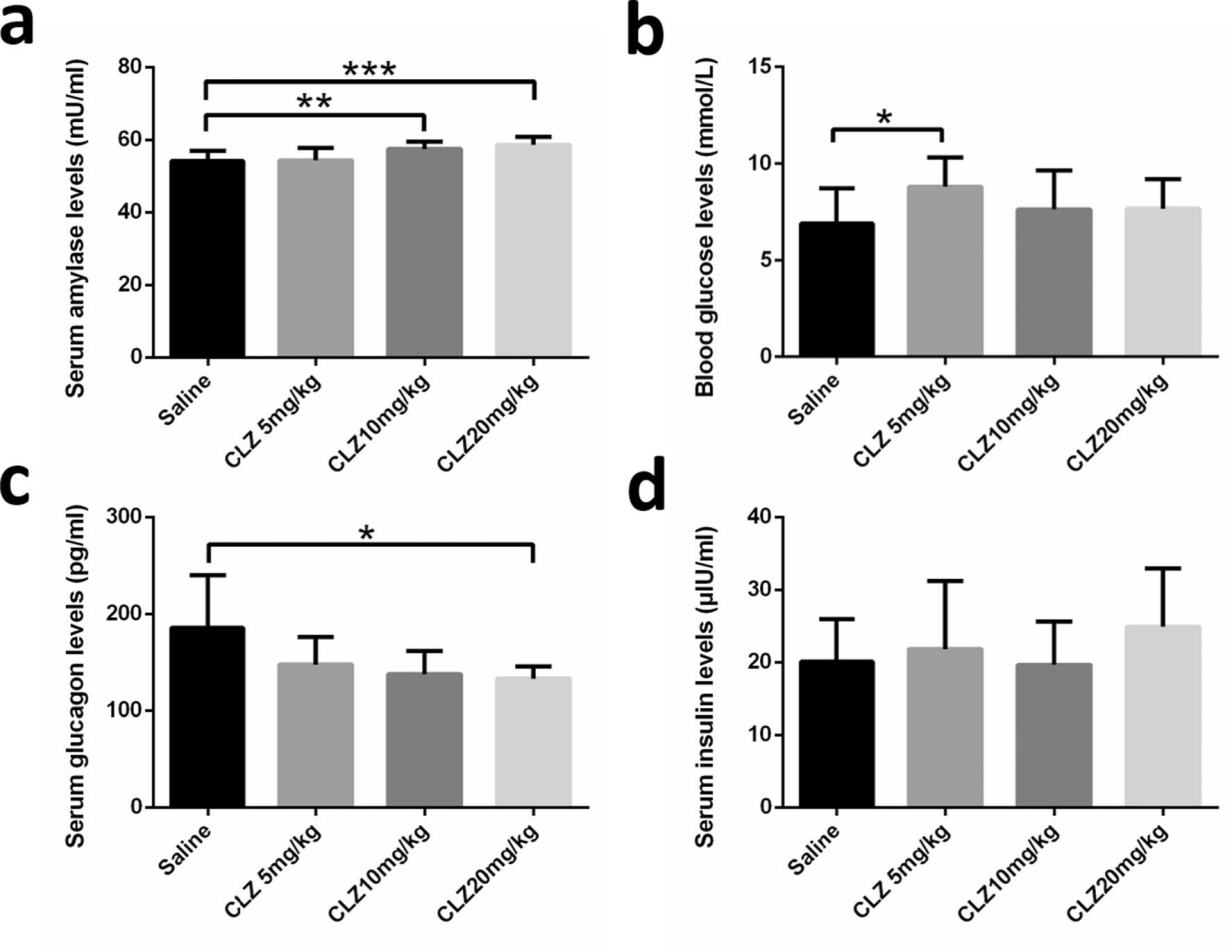
Effect of clozapine on **a** serum amylase, **b** blood glucose, **c** serum glucagon, and **d** serum insulin levels in groups of mice receiving daily intragastric administration of the indicated doses of clozapine for 14 days. A single asterisk denotes statistical difference (*P *< 0.05) compared to the saline group; double and triple asterisks denote significant differences (*P* < 0.01 and *P* < 0.001, respectively) compared to the saline group. Variables are expressed as the means ± standard deviation (*n* = 12)

We evaluated the effect of clozapine on serum amylase, fasting blood glucose, serum insulin, and serum glucagon levels after the animal experiment. As shown in Fig. 2a, serum amylase levels changed significantly after treatment with clozapine (*F* = 7.890, df = 3, *P* = 0.000). Serum amylase levels were significantly increased in the 10 and 20 mg/kg clozapine groups (*P* < 0.01 and *P* < 0.001) compared with the saline group, while the difference in serum amylase levels between the 5 mg/kg clozapine group and the saline group was not statistically significant. Following clozapine treatment, there were no significant changes in fasting blood glucose levels (*F* = 2.260, df= 3, *P* = 0.096). Post hoc tests indicated that blood glucose was significantly increased at the 5 mg/kg group compared to the saline group (*P *< 0.05) (Fig. 2b). However, there was a significant groups effect for serum glucagon levels after treatment with clozapine (*F* = 5.796, df = 3, *P* = 0.002). Post hoc tests indicated that glucagon was significantly decreased at the 20 mg/kg group compared to the saline group (*P* < 0.05) (Fig. 2c). Serum levels of insulin not changed after treatment with clozapine (*F* = 1.185, df = 3, *P* = 0.3270) (Fig. 2d).

#### The effect of clozapine on proinflammatory cytokines levels in the peripheral blood

**Fig. 3:**
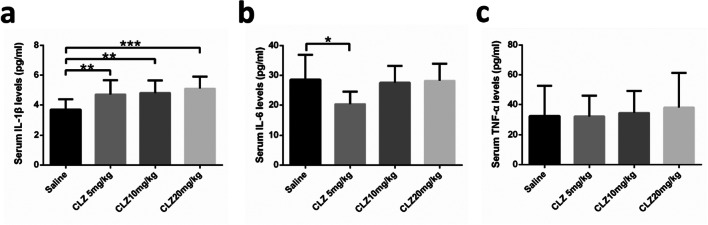
Effect of clozapine on serum **a** IL-1β, **b** IL-6, and **c** TNF-α levels in groups of mice receiving daily intragastric administration of the indicated doses of clozapine for 14 days. Single asterisk denotes statistical difference (*P* < 0.05) compared to the saline group; double and triple asterisks denote significant differences (*P* < 0.01 and *P* < 0.001, respectively) compared to the saline group. Variables are expressed as the means ± standard deviation (*n* = 12)

To demonstrate whether the effect of clozapine treatment altered immune function, the levels of three proinflammatory cytokines, namely IL-1β, IL-6, and TNF-α, were examined in the circulating blood of mice after the experiment. As shown in Fig. 3a, serum IL-β levels were markedly enhanced after treatment with clozapine (*F* = 6.976, df = 3, *P* = 0.001). Furthermore, the 5 mg/kg and 10 mg/kg groups had significantly increased serum IL-1β levels compared with the saline group (all *P* < 0.01), and the effect was most apparent in the 20 mg/kg group compared to the saline group (*P* < 0.001). Clozapine treatment also affected serum IL-6 levels after treatment with clozapine (*F* = 4.033, df = 3, *P* = 0.013). Post hoc tests indicated that serum IL-6 levels were decreased at the lowest dose (5 mg/kg) group relative to saline group (*P* < 0.05). The difference in serum IL-6 levels between the groups treated with higher doses (10 and 20 mg/kg) and the saline group was not statistically significant (Fig. 3b). Serum TNF-α levels were not significantly different among the four groups of mice after clozapine treatment (*F* = 0.272, df = 3, *P* = 0.845) (Fig. 3c).

#### The effect of clozapine on proinflammatory cytokines expression in pancreatic tissue

**Fig. 4:**
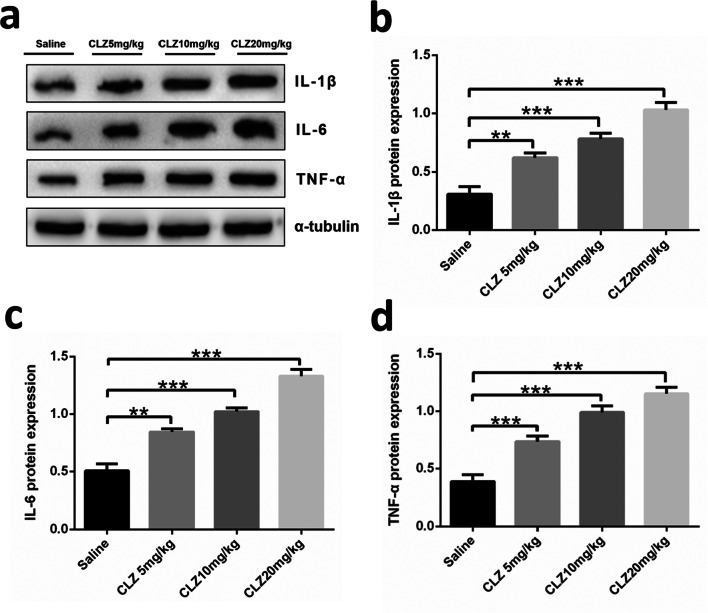
After four groups of mice received daily intragastric administration of the indicated doses of clozapine for 14 days, the protein expression of **b** IL-1β, **c** IL-6, and **d** TNF-α in the mice pancreas was determined by western blot analysis. **a** Quantification of expression normalized to α-tubulin. Double and triple asterisks denote significant differences (*P* < 0.01 and *P* < 0.001, respectively) compared to the saline group. Variables are expressed as the means ± standard deviation (*n* = 6)

**Fig. 5:**
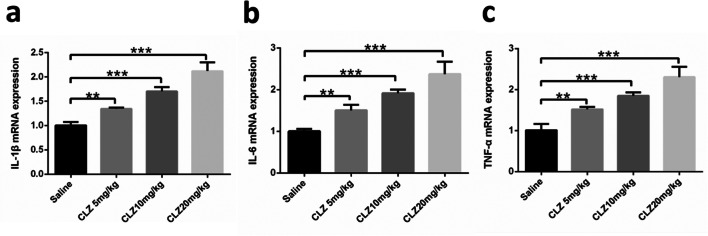
After four groups of mice received daily intragastric administration of the indicated doses of clozapine for 14 days, the mRNA expression of **a** IL-1β, **b** IL-6, and **c** TNF-α in the mice pancreas was determined by qRT-PCR analysis. Double and triple asterisks denote significant differences (*P* < 0.01 and *P* < 0.001, respectively) compared to the saline group. Variables are expressed as the means ± standard deviation (*n* = 6)

The parameters described above are related to pancreatic metabolism. The main functions of the pancreas are to produce exocrine enzymes, including amylase, to aid digestion and to produce endocrine hormones, including insulin and glucagon, to regulate blood glucose (Zhou and Melton 2018). Therefore, we measured proinflammatory cytokines in the pancreatic tissue of mice and determined whether treatment with the indicated doses of clozapine-induced cytokine release. We used western blotting and qRT-PCR to measure the levels of the proinflammatory cytokines IL-1β, IL-6, and TNF-α. As shown in Fig. 4, the protein expression of IL-1β, IL-6, and TNF-α in the pancreas was markedly enhanced after treatment with clozapine [(*F* = 184.349, df = 3, *P* = 0.000), (*F* = 332.945, df = 3, *P* = 0.000), and (*F* = 219.696, df = 3, *P* = 0.000)], and there was a statistical difference between the clozapine 5, 10, and 20 mg/kg groups and the saline group (all *P* < 0.01). As shown in Fig. 5, the mRNA expression of IL-1β, IL-6, and TNF-α was largely consistent with the expression of the respective proteins [(*F* = 125.620, df = 3, *P* = 0.000), (*F* = 70.350, df = 3, *P*= 0.000), and (*F* = 74.281, df = 3, *P* = 0.000)].

### Clinical trial

To demonstrate the role of proinflammatory cytokines in glycometabolism disorders in humans taking clozapine, we recruited a group of male schizophrenic patients taking clozapine and detected these indicators in the peripheral blood to determine differences between the patients and age- and sex-matched healthy controls. As shown in Table 2, significant increases in BMI and glucose levels were observed in clozapine-treated patients compared with controls (*P* = 0.03 and *P* = 0.03). Meanwhile, plasma insulin, glucagon, and amylase levels showed no differences between the two groups. We used the cytometric bead array method to measure the levels of the proinflammatory cytokines IL-1β, IL-6, and TNF-α in clinical subjects’ peripheral blood. In the clozapine-treated group, the proinflammatory cytokines IL-1β, IL-6, and TNF-α were higher compared with the control group (all *P* < 0.001). Pearson correlation analysis revealed a significant positive correlation between glucose and the proinflammatory cytokines IL-6 and TNF-α (*P* = 0.01 and *P* = 0.04) in the clozapine-treated group. In addition, clozapine daily dosage showed a significant positive correlation with the proinflammatory cytokines IL-1β and IL-6 (*P* = 0.02 and *P* = 0.02) in the clozapine group (Table 3). The control group did not demonstrate any significant correlation between glucose metabolism-related parameters and proinflammatory cytokines levels (data not shown).

**Table 2:** Demographic data, glucose metabolism-related parameters, and proinflammatory cytokines levels of patients treated with clozapine and healthy controls

	Clozapine-treated patients (*n* = 20)	Healthy controls (*n* =20)	*t*	*p*
Age (years)	43.60 ± 6.70	43.50 ± 6.25	0.05	0.96
BMI (kg/m^2^)	26.35 ± 3.60	24.09 ± 2.43	2.32	***0.03***
Dosage (mg/day)	238.75 ± 83.70			
Duration (weeks)	19.57 ± 10.68			
Amylase (U/L)	51.20 ± 12.22	48.40 ± 7.91	0.86	0.36
Glucose (mmol/L)	6.21 ± 1.97	5.14 ± 0.88	2.23	***0.03***
Insulin (mU/L)	8.73 ± 4.55	8.19 ± 3.78	0.41	0.69
Glucagon (pg/ml)	193.06 ± 160.24	279.64 ± 235.87	−1.36	0.18
IL-1β (pg/ml)	0.91 ± 0.28	0.58 ± 0.17	4.59	***0.00***
IL-6 (pg/ml)	1.59 ± 0.87	0.65 ± 0.28	4.64	***0.00***
TNF-α (pg/ml)	0.42 ± 0.22	0.19 ± 0.19	3.49	***0.00***

*p* value of the two-sample *t*-test

**Table 3:** The correlation of proinflammatory cytokines levels with the clozapine dosage/duration and glucose metabolism-related parameters in the clozapine-treated group

Variables	IL-1β		IL-6		TNF-α	
	*r*	*p*	*r*	*p*	*r*	*p*
Dosage	0.53	***0.02***	0.53	***0.02***	0.22	0.36
Duration	0.31	0.19	−0.15	0.54	0.50	0.33
BMI	0.09	0.69	0.26	0.26	0.25	0.28
Amylase	0.21	0.37	0.15	0.52	0.19	0.42
Glucose	0.39	0.09	0.55	***0.01***	0.46	***0.04***
Insulin	0.29	0.21	0.27	0.25	0.35	0.13
Glucagon	−0.27	0.24	−0.29	0.22	−0.42	0.06

*p* value of the Pearson correlation analysis

## Discussion

We first examined the changes in glycometabolism parameters of mice after two weeks of treatment with clozapine. Our results showed that blood glucose levels in mice were affected by clozapine, with increased levels of blood glucose in the group treated with clozapine 5 mg/kg. We found that serum glucagon levels decreased with increasing clozapine doses, but there were no changes in serum insulin levels. Insulin and glucagon are the predominant hormones secreted by the pancreas, and their interplay has a pivotal role in the regulation of glucose homeostasis (Röder et al. 2016). We monitored the weight of the mice before daily administration, and our results indicated that mice body weight significantly decreased after receiving daily intragastric administration of clozapine at doses of 10 and 20 mg/kg for 14 days. Endocrine alterations induced by one-time acute administration of clozapine 2.5–20 mg/kg have been observed in animal models (Dwyer and Donohoe 2003; Savoy et al. 2010). However, at similar doses in subchronic or chronic rodent models, clozapine did not cause typical obesity, hyperglycemia, or insulin resistance. Cheng reported that, after 30 days of clozapine administration (2, 10 mg/kg), male mice showed no significant changes in blood glucose, and both dose groups experienced significant weight loss (Cheng et al. 2005). Cooper reported that, in female rats, treatment with clozapine for 20 days consecutively (2, 4 mg/kg, bid) did not affect blood glucose or insulin. The researchers also tested a higher dose of clozapine (6, 12 mg/kg, bid) and found significant weight loss on day 14 (Cooper et al. 2008). The results of our glycometabolism indicators were consistent with the results of Cheng and Cooper’s clozapine chronic and subchronic rodent models. We found that the serum glucagon of mice decreased after administration of 20 mg/kg clozapine for 14 days. The main function of glucagon is to raise blood glucose, and the excessive dose may cause damage to the pancreatic endocrine glands, thereby reducing the secretion of glucagon (Liu et al. 2017). This may explain why the increased blood glucose appeared only in the low-dose group, while the increase in blood glucose levels in the higher-dose group was not statistically significant. It also indirectly supports the previous study that acute glucose metabolism disorder caused by clozapine is mediated by increased glucagon secretion (Nagata et al. 2018).

Interestingly, we found that the serum amylase level of mice increased with the clozapine dose. Amylase is a digestive enzyme that is secreted by the exocrine pancreas and regulates energy consumption and metabolism (Röder et al. 2016). To our knowledge, there is no research on the effects of clozapine on amylase in animals. In the study of olanzapine, which is also an atypical antipsychotic drug and similar to clozapine in structure and receptor profile, chronic oral administration of olanzapine caused rats to show a significant increase in blood amylase (Shah et al. 2016). One of the side effects of clozapine is pancreatitis, which mainly manifests as hyperamylasemia (Lally et al. 2018). An earlier report found transient increases in peripheral blood amylase levels in patients undergoing chronic titration of clozapine (Bergemann et al. 1999). This is consistent with our results of the serum amylase level of clozapine-treated mice.

On the one hand, our clinical results concerning glucose metabolism parameters showed that the BMI and blood glucose of patients taking clozapine were higher than those of healthy controls, which is consistent with the results of previous clinical reports (Lindenmayer et al. 2003). Most clinical trials have shown that clozapine is the most likely to causes obesity, glycemia, and other metabolic disorders among second-generation antipsychotics (Henderson et al. 2000; Hirsch et al. 2017), although recent clinical observations have indicated that clozapine treatment can cause impaired glucose regulation independent of weight gain (Beumer et al. 2012; Siafis et al. 2018). This is strange because impaired blood glucose regulation is generally thought to be secondary to factors such as obesity (Newcomer 2005). On the other hand, the discrepancy of antipsychotic drug-induced metabolic disorders in preclinical models is a major issue of translational neuropharmacology. The effects of antipsychotics on weight gain and metabolic problems are clinically evident. Unlike humans, in the rodent model, this phenomenon seems to be more significant in acute rather than chronic antipsychotic drugs (Benarroch et al. 2016). However, research focusing on clozapine has not yet elucidated whether the induced proinflammatory cytokines participate in the development of metabolic disorders or are secondary to this process.

Given that there is enough evidence of a link between metabolic disorders caused by clozapine and early immune responses, assessing the levels of proinflammatory cytokines may help to detect metabolic problems early (Alvarez-Herrera et al. 2020). In our animal experiment, the serum level of IL-β and the expression of IL-β in pancreatic tissue were markedly enhanced after treatment with clozapine, which is in line with the results of our clinical trials. A significant increase in plasma IL-1β was observed in clozapine-treated patients compared with healthy controls. Our results are also consistent with those of a clinical study with a large sample (O’Connell et al. 2014). Our clinical correlation analysis results also showed that IL-1β levels were positively correlated with the daily dosage of clozapine. The cytokine IL-1β is a central mediator of inflammation that is critical for defense against infections and injuries and is also associated with glycometabolism disorders (Dinarello 2011). It is well documented that increased circulating IL-1β is a hallmark of the chronic, low-grade inflammation associated with glycometabolism disorders. The circulating levels of IL-1β in obese individuals with prediabetes are similar to the levels in those with overt diabetes (Febbraio 2014). Further studies have shown that treatment of type 2 diabetes patients with an IL-1 receptor (IL-1R) antagonist diminishes blood glucose and systemic inflammatory markers (Larsen et al. 2007). A mice model of gestational diabetes has also found that inhibiting the IL-1β signaling pathway improves glycemia (Schulze et al. 2020). The findings reported above indicate that IL-1β can be used as an early predictor of glycometabolism disorders. It also explains the development process from the early inflammatory state to the occurrence of metabolic disorders (Dror et al. 2017). This gives further support to the concept that inflammatory mediators control metabolic disease and also take part in physiological metabolic regulation, a concept often called “immunometabolism” (Mathis and Shoelson 2011).

As the physiologic role of the immune system in regulating glycometabolism is not limited to IL-1β, we also examined other components of the immune system that might be involved in this process. The results of our clinical trials showed that the plasma IL-6 and TNF-α levels of patients taking clozapine were significantly higher than healthy controls. This is consistent with the results of previous clinical studies of clozapine (Hinze-Selch et al. 2000; Monteleone et al. 1997), but findings of decreased levels or no effects have also been reported (Himmerich et al. 2011; O’Connell et al. 2014). Our clinical correlation analysis results also showed that, in patients treated with clozapine, IL-6 level was positively correlated with clozapine daily dosage and glucose level. In a previous study, compared with healthy controls, there was a significant positive correlation between serum IL-6 and BMI in patients (Klemettilä et al. 2014). Recent studies have also found that IL-6 has an inflammatory regulatory effect on SGAs-induced metabolic disorders (Fang et al. 2019). Our correlation analysis showed that TNF-α level was positively correlated with glucose level in patients treated with clozapine. In previous studies on clozapine, the elevated level of TNF-α has always been related to metabolism. A study found elevated level of TNF-α in chronic schizophrenia patients with clozapine-associated obesity (Klemettilä et al. 2014). Another study found a correlation between increases in TNF-α level and BMI during initial clozapine treatment (Kluge et al. 2009). Therefore, TNF-α may be an early sensitive marker of metabolic disorders during clozapine therapy (Baptista and Beaulieu 2002).

We also found that clozapine did not affect TNF-α in the peripheral blood of mice, while IL-6 was reduced to a certain extent in the low-dose clozapine group. Since the IL-6 level continued to increase in the higher-dose clozapine group, and decreases in the level of IL-6 in the low-dose group were not apparent, we considered that this small difference in the low-dose group might be a false-positive. In fact, the effect of clozapine on IL-6 and TNF-α was inconsistent in vivo in clinical trials in patients, and the effects of clozapine on peripheral blood IL-6 and TNF-α also seem to be elusive in vivo in animal experiments (Røge et al. 2012). In our previous research, clozapine (1 mg/kg) administration for two consecutive weeks could up-regulate rat serum IL-6 levels but had no effect on serum TNF-α (Liu et al. 2015). Some evidence suggests that the cytokines secretion upon chronic administration of clozapine (20 mg/kg/day) is gender dependent: serum IL-6 and TNF-α increased in female rats, but only serum TNF-α level increased in male rats (Nikolić et al. 2017). Additionally, acute administration of clozapine inhibited the increase of serum TNF-α and IL-6 in lipopolysaccharide-treated mice (Sugino et al. 2009). Although we did not find a consistent trend of clozapine causing IL-6 and TNF-α changes in the peripheral blood of mice, we did find that the expression of IL-6 and TNF-α levels were increased in the pancreatic tissue of mice. Research has indicated that long-term olanzapine may induce glucose metabolic disorders by activating a peripheral inflammatory response, upregulating proinflammatory cytokines in plasma and adipose tissue (Li et al. 2019). However, the impact of clozapine on proinflammatory cytokines in different tissues has received limited attention (Røge et al. 2012). There are no reports of proinflammatory cytokines expression in pancreatic tissue after clozapine treatment in animals. Our results in mice are consistent with the study of a streptozotocin-induced diabetes rat model (Hsiao et al. 2019), wherein increased levels of the proinflammatory cytokines IL-6, TNF-α, and IL-1β were found in diabetic rat pancreatic tissue. One possible explanation is that a unique mechanism of clozapine can modulate inflammatory function with idiosyncratic patterns, suggesting that the immunomodulatory effects of this agent may play a role in the development of glycometabolism disorders (Røge et al. 2012).

This research has several limitations. First, the clinical trials were limited by their small sample size, but the inclusion of age- and sex-matched controls somewhat increased the power of this small study. Second, the clinical study had a cross-sectional design, which prevented us from making conclusions about causality. Third, we did not establish a schizophrenia model in mice; therefore, the animal experiments do not completely correspond to the clinical trials. Fourth, the influence of schizophrenia disease itself on the detection parameters has not been ruled out in the clinical study. Fifth, the results of glycometabolism-related parameters and proinflammatory cytokine indicators in animal experiments and clinical trials are not completely consistent. This further illustrates that the transformational discrepancy of preclinical models is a major problem in psychopharmacology. However, to our knowledge, this is the first study to combine clinical trials and animal experiments to explore the association between the mechanism of clozapine-induced metabolic disorders and immunomodulatory effects. Our research was incomplete and preliminary, and further research should be carried out to improve and verify our findings.

## Conclusion

Our results show that murine serum glucagon levels were significantly decreased, serum amylase and proinflammatory cytokine IL-β levels markedly enhanced after clozapine administration. Clozapine reliably increased proinflammatory cytokines (IL-β, IL-6, and TNF-α) expression in murine pancreatic tissue. BMI, blood glucose, and proinflammatory cytokines (IL-β, IL-6, and TNF-α) levels increased significantly in clozapine-treated patients compared with healthy controls. In patients taking clozapine, the levels of peripheral blood proinflammatory cytokines were positively correlated with glucose and clozapine daily dosage. In conclusion, our animal experiments and clinical trials have shown that clozapine has an effect on glycometabolism-related parameters, and there is also clear evidence that clozapine has a regulatory effect on immune-related proinflammatory cytokines. However, the role of proinflammatory cytokines in clozapine-induced glycometabolism disorders is not as straightforward as may be assumed. Future studies should conduct comparative experiments with large samples in animals and humans, which may represent a way to reduce the harm caused by glycometabolism disorders mediated by clozapine.
